# Validating faecal glucocorticoid metabolite analysis in the Virunga mountain gorilla using a natural biological stressor

**DOI:** 10.1093/conphys/cow029

**Published:** 2016-08-30

**Authors:** W. Eckardt, T. S. Stoinski, S. Rosenbaum, M. R. Umuhoza, R. Santymire

**Affiliations:** 1 The Dian Fossey Gorilla Fund International, Atlanta, GA 30315, USA; 2 Institute for Mind and Biology, University of Chicago, Chicago, IL 60637, USA; 3 Davee Center for Epidemiology & Endocrinology, Lincoln Park Zoo, Chicago, IL 60614, USA

**Keywords:** Ape, circadian pattern, faecal sample, interaction, lag time

## Abstract

The continued degradation of primate habitat worldwide is forcing many primate populations into small protected forest islands surrounded by high-density human populations. One well-studied example is the critically endangered mountain gorilla (*Gorilla beringei beringei*). Decades of monitoring and research on Rwanda's mountain gorillas offer a unique opportunity to use non-invasive endocrine analysis to address pressing questions about the conservation of this endangered population. The aims of our study were as follows: (i) to validate field and laboratory methods for assessing stress through faecal glucocorticoid metabolite (FGM) analysis using inter-social unit interactions as a natural stressor; (ii) to determine the excretion lag times between interactions and detectable stress response in faeces; and (iii) to determine whether there are circadian patterns of FGM excretion. We collected ~6000 faecal samples from 127 known gorillas in 10 habituated groups, monitored by the Dian Fossey Gorilla Fund's Karisoke Research Center over 21 months in 2011 and 2012. Extracted FGMs were measured using a cortisol enzyme immunoassay (R4866; C. J. Munro). Results revealed cause–effect relationships between inter-unit interactions and increased FGMs (relative to individual pre-event samples) between 20 and 140 h after interactions, with the peak most often occurring on day 3. There was no evidence of circadian patterns in FGM concentrations, as previously shown in many species with long gut passage times. However, baseline FGM concentrations were lower in adult males than in adult females, and variation was associated with the collection month, indicating possible seasonal variation. This study provides a biologically validated, field-friendly faecal hormone metabolite extraction and laboratory enzyme immunoassay analysis method for non-invasive monitoring of adrenocortical activity in Virunga mountain gorillas. The methods are useful for future evaluation of a variety of environmental and human-induced potential stressors in this critically endangered population.

## Introduction

‘Stress’ is a widely discussed topic because of its potential negative health effects, especially when it is chronic. Physiological stress occurs when stimuli lead to ‘allostatic overload’ (McEwen and Wingfield, 2003); that is, energetic demands to maintain or re-establish homeostasis exceed available energy supplies. However, allostatic overload can also occur when an organism experiences social disruptions or conflicts even when energy balance is neutral or positive. In mammals, allostatic overload causes a host of physiological responses, including the release of a cascade of hormones by glands constituting the hypothalamic–pituitary–adrenal axis. The adrenal glands secrete glucocorticoids (GCs), which then signal back to the hypothalamic–pituitary–adrenal axis, creating a negative feedback loop (Romero, 2004). Glucocorticoid secretion leads to a rapid increase in gluconeogenesis, which mobilizes energy reserves to respond adaptively to stressors through a ‘fight-or-flight’ response (reviewed by Sapolsky *et al*., 2000). Prolonged allostatic overload disrupts the negative feedback mechanism and can lead to deleterious health outcomes for the individual (Sapolsky *et al*., 2000; McEwen and Wingfield, 2003; Romero, 2004; Tarlow and Blumstein, 2007; Tort and Teles, 2007). Negative effects are variable and include reproductive inhibition, immune system suppression, neuron loss and impaired cognitive function, and impaired growth and development (Sapolsky *et al*., 2000; McEwen and Wingfield, 2003; Charmandari *et al*., 2005; Tort and Teles, 2007). Such detrimental effects on individual organisms and, potentially, populations can threaten the survival of endangered species.

The integration of hormone analysis into wildlife conservation research is a prevailing and widely accepted approach (Ganswindt *et al*., 2012). Hormones such as GCs provide precise measures of adrenocortical activity in captive and wild animal populations and, thus, crucial information for addressing important conservation questions and developing effective interventions (Wielebnowski and Watters, 2008; Ayres *et al*., 2012; Ganswindt *et al*., 2012). For example, Ayres and colleagues (2012) assessed a combination of triiodothyronine (an indicator of nutritional stress) and GC in endangered killer whales (*Orcinus orca*). Their analyses revealed that a marked reduction in availability of the whales’ key prey, Chinook salmon (*Oncorhynchus tshawtscha*), was related to the decline of the whale population, as opposed to psychological stress associated with noise disturbance from growing vessel traffic.

Techniques for measuring GC metabolites in faeces have been used to monitor physiological stress responses to environmental and social changes in wild animal populations (Ganswindt *et al*., 2012). This non-invasive method allows for long-term physiological monitoring in natural settings with minimal animal disturbance. It is typically used concurrently with other biological measures, such as behaviour, biotic (e.g. predator pressure, food availability) and abiotic (e.g. temperature, rainfall) environmental factors (Ayres *et al*., 2012), anthropogenic disturbances (Tarlow and Blumstein, 2007), and demography and health (e.g. parasite load; Muehlenbein, 2006). Faeces are particularly suitable for long-term monitoring and capturing seasonal and chronic stressors. They contain pooled hormone metabolite concentrations rather than a discrete amount of hormone secreted at a specific time, as occurs in blood samples (Millspaugh and Washburn, 2004).

Faecal hormone metabolite extraction at field sites offers multiple advantages over shipping preserved or dried faecal samples to laboratories for analysis (Ziegler and Wittwer, 2005; Santymire and Armstrong, 2010; Palme *et al*., 2013). For example, using preservatives or drying samples increases the risk of degrading steroid metabolites (see Santymire and Armstrong 2010; Palme *et al*., 2013). Shipping small vials containing extracted hormones rather than preserved faecal samples also reduces transportation costs, eliminates the complications of shipping flammable preservatives and, typically, does not require import permission for countries where analysis laboratories are based. Developing extraction laboratories at field sites also contributes to capacity building of local scientists.

Although it has many advantages, faecal hormone analysis measures hormone metabolites, not the native hormone, so it requires extensive biological and biochemical validation before application. Each species and hormone must be validated individually owing to inter-specific differences in steroid hormone metabolism and route before secretion into faecal matter (Reimers *et al*., 1981; Heistermann *et al*., 2006). Once validated, faecal hormone metabolite analysis can be used to evaluate environmental and social variables that may be limiting the maintenance or growth of endangered populations. Such information facilitates effective conservation policy.

The Virunga mountain gorilla (*Gorilla beringei beringei*) is a critically endangered species; only 880 individuals remain in two isolated populations (Gray *et al*., 2013; Roy *et al*., 2014). In the protected area they inhabit, multiple conservation management strategies are used; these include regular patrols to target persistent illegal activities, gorilla habituation for eco-tourism, and long-term research and health monitoring combined with an on-site veterinary intervention programme. These collective long-term conservation efforts have resulted in a steady population increase (Gray *et al*., 2010, 2013). Although mountain gorillas are a unique success story in ape conservation, this success is accompanied by challenges. There is an ever-growing human presence in the forest—specifically near the gorillas—and the animals are experiencing ongoing changes in population dynamics as their numbers increase in a space-restricted forest island. Changes in the population dynamics include an up to 6-fold increase of inter-social unit interactions in some areas (Caillaud *et al*., 2014). Inter-unit interactions can be very violent and cause severe injuries or death of involved males or dependent offspring (Fossey, 1984; Watts, 1989; Sicotte, 1993). To ensure a sustainable, healthy population, non-invasive assessments of gorilla health are an essential aspect of long-term monitoring programmes. Ongoing monitoring of the gorillas and their habitat allows for the study of hormone patterns and their relationship to myriad social and environmental factors. Given that samples can be obtained from individually identified gorillas, analyses can control for factors such as sex (Goymann, 2012), age (Boscaro *et al*., 1998), social status (Goymann and Wingfield, 2004; Gesquiere *et al*., 2011; Markham *et al*., 2013) and reproductive status (Hoffman *et al*., 2010).

Faecal hormone metabolite analysis has been validated for both captive and wild western lowland gorillas (*Gorilla gorilla gorilla*; captive: Heistermann *et al*., 2006; [Bibr cow029C40][Bibr cow029C41]; Shutt *et al*., 2012; Jacobs *et al*., 2014; wild: Shutt *et al*., 2012). Adrenocorticotrophic hormone challenge tests validated GC analysis techniques in zoo-housed western lowland gorillas (Heistermann *et al*., 2006; Nizeyi *et al*., 2011a; Shutt *et al*., 2012; Jacobs *et al*., 2014). Performing an adrenocorticotrophic hormone challenge in mountain gorillas is impractical, because there is not an established captive population. However, comparing GC concentrations of samples collected before and after natural biological stressors in wild populations provides an alternative approach for validating a GC assay. Faecal glucocorticoid metabolite (FGM) analysis from wild mountain gorillas living in Bwindi Impenetrable Forest National Park in Uganda was biologically validated using samples from before and after gorillas raided crops and were chased back into the forest by local people (Nizeyi *et al*., 2011a). Although characterization of stress physiology of the Bwindi mountain gorilla population provides useful insights for both populations, this population differs from the Virunga population in several key aspects, including diet and altitudinal range (Watts, 1984; Ganas *et al*., 2004; Harcourt and Stewart, 2007). These differences may affect hormone metabolism (Beehner and McCann, 2008; Goymann, 2012), so additional Virunga population-specific validation is necessary.

The specific objectives of our study were as follows: (i) to validate standardized field and laboratory methods to characterize the stress physiology of Virunga mountain gorillas using FGMs for analysis and inter-social unit interactions; (ii) to determine the excretion lag times between interaction and detectable stress response in faeces; and (iii) to determine circadian patterns of FGM excretion. By developing non-invasive methods to characterize stress physiology in the largest population of this endangered species, we can learn more about how environmental pressures, including anthropogenic, social and ecological change, are impacting the health and long-term population success of this critically endangered species.

## Materials and methods

### Study site and animals

Between April 2011 and December 2012, behavioural data and faecal samples were collected on 127 known individual Virunga mountain gorillas monitored by the Dian Fossey Gorilla Fund's Karisoke Research Center (KRC). The animals lived in 10 social units in Volcanoes National Park, Rwanda. Volcanoes National Park comprises the Rwandan portion of the Virunga massif, a montane cloud forest ranging from 2300 to 4500 m altitude, which is shared with Democratic Republic of Congo and Uganda. Temperatures are mild year round. Rainfall is bi-modally distributed, with a long and short wet season lasting from September to December and March to May, respectively (Mehta and Katee, 2005). The principal dry season lasts 2–4 months between May and September, whereas the short dry season from January to March is characterized by reduced rain (Mehta and Katee, 2005).

### Terminology

Throughout, we refer to animals by the established names for their appropriate age/sex class. ‘Silverbacks’ are adult males age ≥12 years; ‘blackbacks’ are post-adolescent males age 8–11 years; adult females are those age ≥8 years; subadults are animals of either sex between 6 and 8 years old; and infants are animals ages 0–3.5 years (Williamson and Gerald-Steklis, 2001; Weber and Vedder, 1983; Fletcher, 1994; Watts and Pusey, 1993). Males living in groups with multiple silverbacks have strong dominance hierarchies (e.g. Stoinski *et al*., 2009). We refer to the male at the top of the dominance hierarchy as the dominant male, and any male second ranked or lower as subordinate. Adult males on their own without a social unit are referred to as solitary silverbacks.

Inter-social unit interactions typically involve ritualized behaviours that can either be performed individually or simultaneously/in quick succession (e.g. vocalizing while engaging in a physical movement). These are usually, but not always, an excited performance by subadult or adult males used to intimidate rivals. When behaviours are performed simultaneously or in quick succession, they are referred to as ‘displays’. Behaviours that may be used individually or in displays include strut-stances (a stiff-appendage posture), chest-beating (alternately hitting the pectoral muscles with both fists), smashing/dragging plants, pounding the ground, wagging the head and/or symbolic feeding (nipping off a leaf and holding it between lips before spitting it out; Fossey, 1983; Fletcher, 1994). Vocalizations performed during displays specifically and interactions more generally include screaming, which is an aggressive vocalization, or hooting, which is often done simultaneously with chest-beating (Fossey, 1972).

### Faecal collection and storage

Faecal samples were collected by KRC field staff between 07.15 and 16.00 h. We aimed to collect one baseline hormone sample per gorilla per week, and *ad libitum* samples from all gorillas affected by inter-unit social interactions for 5 days consecutively post-event (hereafter referred to as event samples). Baseline samples refer to samples that were collected before any event, and ≥6 days after any observed event. Immediately after defecation, subsamples were taken from any section of the faeces, because the distribution of glucocorticoids in mountain gorilla faeces is relatively even (Nizeyi *et al*., 2011b). Small plastic bags were used as gloves to collect and transport the samples. Samples contaminated by urine or exposed to water were not collected, because both factors may affect FGM concentrations (Wasser *et al*., 1998; Washburn and Millspaugh, 2002; Ganswindt *et al*., 2010). Each sample was labelled with the collection date and time and the gorilla's identification code. Samples were kept on ice packs once they reached a car for transport to the KRC laboratory (~1–6 h after collection).

Upon arrival at the laboratory, samples were stored at −20°C and sample label details were noted in a logbook, including the time of storage in the freezer. Samples were typically frozen at ~15.00 h (~5–15 h after collection). A delay in faecal sample freezing can affect the FGM concentration, which may be influenced by environmental conditions and bacterial enzymes (Möstl *et al*., 2005, 1999; Palme *et al*., 2005). Previous reports from the Bwindi mountain gorilla population have shown that FGMs are stable in their natural environment up to 60 h post-defecation (Nizeyi *et al*., 2011b). Note that no information is available about faecal exposure to rain, but given Bwindi's rainy environment (Nizeyi *et al*., 2011b) and that samples for the study by Nizeyi and colleagues (2011b) were deliberately left in the forest environment for resampling for several days, some or most samples were almost certainly exposed.

Three factors suggest that FGMs in the present study should have remained stable for the 5–15 h lag between collection and freezing. First, the annual mean temperatures in the Virungas are lower than those of the section of Bwindi where Nizeyi and colleagues (2011b) collected their samples (Ganas *et al*., 2004), meaning that sample degradation should be slower. Second, none of the faecal material we used was exposed to water (which again may degrade samples), whereas samples in the previous study probably were. Finally, the time from defecation to freezing was randomly distributed amongst the samples, so although this may have added noise to the data, it should not create systematic bias. After approximately 2–3 months of frozen storage, FGMs were extracted from wet faeces in KRC's laboratory by M.R.U. using the faecal hormone extraction protocol described previously by Santymire and Armstrong (2010). This field protocol has been used for other wild apes (Murray *et al*., 2013).

### Field hormone extraction method and extract storage

Each faecal sample was thawed to room temperature and mixed in the plastic bag. We then weighed out 0.5 ± 0.02 g of wet faecal material free from plant parts and other debris, and placed it in the bottom of a labelled 16 mm × 125 mm polypropylene tube. The weight of each sample, label details and any unusual observations were noted on a recording sheet. We then added 5 ml of 90% ethanol:distilled water, and homogenized the sample (OMNI International homogenizer, Kennesaw, GA, USA) for 1 min. The resulting mixture was poured through a funnel-shaped filter paper (VWR, 11 cm diameter, cut in half and rolled) into a clean identical tube. This filtrate resulted in a 1:5 sample dilution in ethanol. Two 1 ml aliquots were pipetted into two new 12 mm × 75 mm polypropylene tubes that were labelled with a sample code, gorilla name, collection date and collection time. Both aliquots were then placed in a rack in a sunny location (when possible) until completely dry (approximately 1–2 weeks). During extreme rainy periods, we used a hair dryer to apply low heat to the hormone extracts (e.g. Loeding *et al*., 2011; Jacobs *et al*., 2014) for ~15 min each day to avoid moulding. Dry samples were capped and stored at −20°C until transport to the Davee Center for Epidemiology and Endocrinology at Lincoln Park Zoo (Chicago, IL, USA) for hormone analysis.

### Cortisol enzyme immunoassay

The dried hormone extracts were reconstituted with 1 ml phosphate-buffered saline following established protocols (Murray *et al*., 2013). The FGMs were quantified using a cortisol enzyme immunoassay (EIA; provided by C. J. Munro, University of California Davis, Davis, CA, USA) employing horseradish peroxidase (1:8500 dilution) ligands and polyclonal antiserum (R4866; 1:20 000 dilution) with previously described methods (Loeding *et al*., 2011). The cortisol EIA cross-reactivities were previously published (Young *et al*., 2004): cortisol, 100%; prednisone, 6.3%; corticosterone, 0.7%; 21-deoxycorticosterone, 0.5%; progesterone, 0.2%; pregnenolone, 0.1%; androstenedione, 0.1%; dehydroisoandrosterone-3-sulfate, 0.1%; estradiol-17β, 0.1%; estriol, 0.1%; cholesterol, 0.1%; prednisolone, 9.9%; cortisone, 5.0%; deoxycorticosterone, 0.3%; 11-desoxycortisol, 0.2%; 17α-hydroxyprogesterone, 0.2%; 17α-hydroxypregnenolone, 0.1%; testosterone, 0.1%; dehydroepiandrosterone, 0.1%; aldosterone, 0.1%; estrone, 0.1%; and spironolactone, 0.1%.

### Validation of adrenocortical activity

High-performance liquid chromatography previously established the presence of FGM in mountain gorilla faeces (Nizeyi *et al*., 2011a). For the present study, the cortisol EIA was validated in the Virunga mountain gorillas by demonstrating the following: (i) parallelism between binding inhibition curves of faecal extract dilutions (*r* = 0.976); and (ii) significant recovery (>90%) of exogenous cortisol added to faecal extracts (*y* = 1.15*x* + 3.59; *R*² = 0.998). Assay sensitivity was 3.9 pg/well, and intra- and inter-assay coefficients of variation were <10%.

### Biological validation

To validate our methods in wild Virunga mountain gorillas, we used faecal samples from 11 gorillas before and after four independent inter-unit interactions. These are usually aggressive and pose a high risk of infanticide and injury (Fossey, 1984; Watts, 1989; Sicotte, 1993). These individuals and interactions were selected for validation analyses because they had the best faecal collection coverage pre- and post-event. Detailed behavioural observations during all observed interactions were recorded by trained KRC field staff who could identify known individuals.

#### Inter-unit interaction 1

The first interaction took place on 21 December 2011 from 09.00 to 12.00 h and involved two groups, Bwenge (BG) and Inshuti (IG). The two groups contained nine and six gorillas, respectively, each with one silverback and associated females and offspring. The interaction started when both silverbacks exchanged chest-beats while ~300 m apart. Inshuti group moved toward BG until they had visual contact with one another at 09.20 h. The silverback in IG strut-stanced and screamed at BG group. When a nulliparous adult female in IG tried to move toward BG, IG's silverback hit and dragged her. During the 3 h interaction, IG's silverback displayed at BG 28 times. The silverback in BG performed chest-beats three times and moved away from IG continuously, while IG followed slowly. Both groups travelled ~1.5 km during the interaction. At 12.00 h, when the groups were separated by ~400 m, IG's silverback stopped displaying and changed directions, followed by his group. For the biological validation, pre- and post-event faecal samples were used from the six gorillas in IG.

#### Inter-unit interaction 2

The second interaction took place on 17 September 2011 at 10.15 h between Ugenda group (UG) and a solitary silverback, and lasted ~5 min. Ugenda group contained 11 individuals, including two silverbacks. The group was apparently surprised by the sudden appearance of the solitary silverback. The solitary silverback bit an infant female who, along with her mother, lagged behind the main group. The infant's finger was severed in the attack. When the infant screamed, group members ran to the interaction site, led by the subordinate silverback in UG. The subordinate silverback bit the solitary silverback, which ran away shortly thereafter. The dominant and subordinate males continued displaying for another 2 min. The subordinate male sustained a small wound on his neck. Faecal samples before and after the event were retrieved from the injured infant, her mother and the group's subordinate male.

#### Inter-unit interaction 3

The third interaction, on 12 January 2012, involved a solitary silverback and Kuryama group (KG). Kuryama group contained 14 individuals, including two silverbacks. The subordinate male in KG gave a series of 23 hooting vocalizations combined with chest-beats from 08.50 to 09.40 h, while the rest of the group continued normal foraging behaviour. During this period, the dominant male in KG displayed four times. At 10.30 h, the solitary silverback approached to within 10 m of KG and displayed, including strut-stancing and pounding the ground with his fists. The three males faced each other in strut-stance posture. After 15 min, the dominant male moved toward the solitary silverback. He was followed by the subordinate male and a blackback in KG, while the rest of the group remained in place and continued feeding. The solitary silverback retreated quickly, followed closely by the KG males. The KG males displayed at the solitary male, including chest-beating, symbolic feeding, smashing plants and strut-stancing. At 11.00 h, the dominant male and blackback returned to the rest of the group and left the subordinate male alone with the solitary silverback. The two males continued to display at each other. The subordinate male rejoined the group 15 min later, leaving the solitary silverback 250 m behind. The dominant male continued hooting and chest-beating periodically until 12.15 h. In total, the dominant and subordinate males displayed 12 and 32 times, respectively. There was no physical contact observed between any of the participants. Faecal samples were obtained from the dominant male before the interaction and during 5 days consecutively after the interaction.

#### Inter-unit interaction 4

In the fourth interaction, groups KG and IG encountered one another on 11 August 2011 at 11.30 h. Inshuti group's silverback moved downhill towards KG, leaving the IG females and infants ~800 m behind. He moved to ~20 m distance from KG and stood in strut-stance posture, at which point KG's subordinate silverback, a blackback and a subadult male noticed his presence. A series of approaches and retreats between KG's dominant silverback and IG's silverback followed. The blackback and two female infants approached the IG silverback closely, while both of KG's silverbacks stood in front. At 11.55 h, KG's dominant silverback returned to the rest of the group, which by this point was slightly behind, and started feeding. IG's silverback, and the subadult male and blackback from KG continued displaying at one another. At 12.17 h the IG silverback retreated towards his group, which remained uphill ~800 m away. KG moved further downhill away from IG, except for the blackback, who followed IG for another 50 min before rejoining KG. Faecal samples before and after the interaction were obtained from the mother of one of the infants that closely approached IG's silverback.

### Data analysis

#### Calculation of hormonal baseline values of FGM concentrations

As the gorillas are not monitored 24 h per day, it is impossible to record all potentially stressful events that occur. Unsurprisingly, some samples classified as baseline samples revealed high FGM concentrations, which might indicate the occurrence of unobserved stressful events. To control for these values, we used an iterative process that excluded baseline samples with values greater than the mean + 1.5 standard deviations (SD) for each individual gorilla. This process was repeated until all baseline samples were either equal to or less than the mean ± 1.5 SD (Moriera *et al*., 2001).

#### Circadian patterns

In order to determine whether FGM concentrations followed a circadian pattern, we divided baseline samples by collection time into morning (before noon) and afternoon samples. Adult gorillas (≥8 years old) with fewer than three baseline samples in either of the two categories were excluded from this analysis. Collection time was entered as a fixed effect in a linear mixed-effects model, with baseline hormone values as the response variable and gorilla identification nested within social groups as random effects. We also included two potentially confounding variables as fixed effects, namely sex of the adult gorillas (44 females and 28 males) and the collection month, because FGM concentrations in mammals can vary according to sex (Touma and Palme, 2005) and season (Touma and Palme, 2005; Gesquiere *et al*., 2008). These and all other statistical tests were run using R (version 3.1.2; R Core Team, 2015).

#### Time gap between event and elevated FGM concentration

In order to determine excretion lag times between exposure to a stressor and elevated FGM concentrations, we used a larger data set involving 25 inter-unit interactions and 233 samples collected from a total of 34 gorillas 1.8–31.9 years of age (Table 1) 1 week before (pre-event) and up to 10 days after inter-unit interactions. For analysis of the time lag between observed events and elevated FGM concentration, we excluded gorillas without evidence of elevated FGM concentrations following an interaction (284 out of 406 cases). Unaltered post-event FGM concentrations might indicate a lack of interaction-induced stress in some gorillas or insufficient post-event sampling. Seventy-two other cases were omitted because of missing pre-event samples 1 week before the interaction. We ran a linear mixed-effects model, with the lag time in relationship to the interaction event as a fixed effect. These values were divided into pre-event samples, collected before the interaction, and 20 h post-event interval categories starting with >0–20 h and extending up to >200–220 h post-event. The response variable was log-transformed FGM concentrations of the pre-event and post-event samples. Gorilla identity nested within social group identity were entered as random effects.

**Table 1:** Age and sex distribution of the gorillas used in analysis of the time lag between inter-unit interaction events and elevated faecal glucocorticoid metabolite concentrations

Age/sex category	Age range (years)	Females (*n*)	Males (*n*)	Total (*n*)
Full-grown silverback	>15	–	5	5
Young silverback	>12–15	–	2	2
Blackback	>8–12	–	0	0
Adult female	>8.0	16	–	16
Sub-adult	>6.0–8.0	0	0	0
Juvenile	>3.5–6.0	4	3	7
Infant	0–3.5	2	2	4

Age/sex classifications are from Table II of Breuer *et al*. (2009).

## Results

### Baseline sample variation

**Figure 1:**
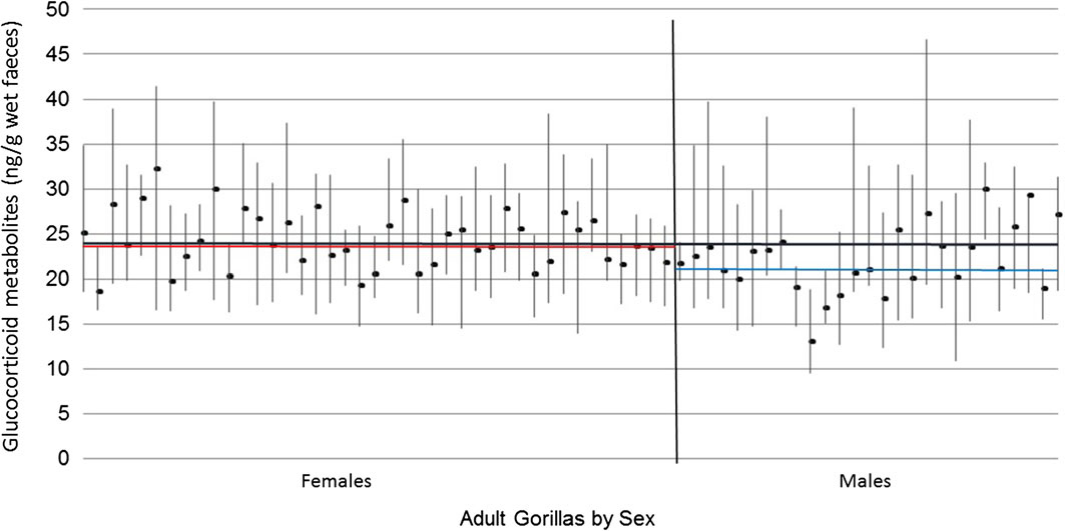
Adult male mountain gorillas (*n* = 28) had lower mean baseline faecal glucocorticoid metabolite concentrations than adult females (*n* = 44; *F* = 13.20, *P* < 0.001, *n* = 1936 faecal samples). Individual points represent the mean ± 1.5 SD for each animal; the vertical black lines are the upper and lower 95% confidence intervals. The blue line represents the mean of the mean ± 1.5 SD for all adult males; the red line represents the mean of the mean ± 1.5 SD for all adult females.

The mean baseline FGM concentration in adult mountain gorillas ranged from 13.0 to 32.2 ng/g wet faeces (range for females, 18.5–32.2 ng/g wet faeces and for males, 13.0–29.9 ng/g wet faeces; Fig. 1).

### Circadian, monthly and sex-specific patterns of FGM concentrations

For baseline samples from adult gorillas, there was no difference between FGM concentrations of samples collected in the morning vs. the afternoon, after controlling for sex and collection month (morning samples, 18.18 ± 4.31 ng/g wet faeces, *n* = 1299; afternoon samples, 18.23 ± 4.15 ng/g wet faeces, *n* = 637; β = 2.16, SE = 1.63, *F* = 1.74, d.f. = 1, *P* = 0.186).

Adult males (*n* = 28) had lower baseline FGM concentrations than adult females (*n* = 44; *F* = 13.20, d.f. = 1; β = 1.69, SE = 0.46, *n* = 1936 samples, *P* < 0.001; Fig. 1). Baseline FGM concentrations varied with the month in which the faecal sample was collected (*F* = 9.19, d.f. = 11; *n* = 1936 samples, *P* < 0.001).

### Biological validation

#### Inter-unit interaction 1

**Figure 2:**
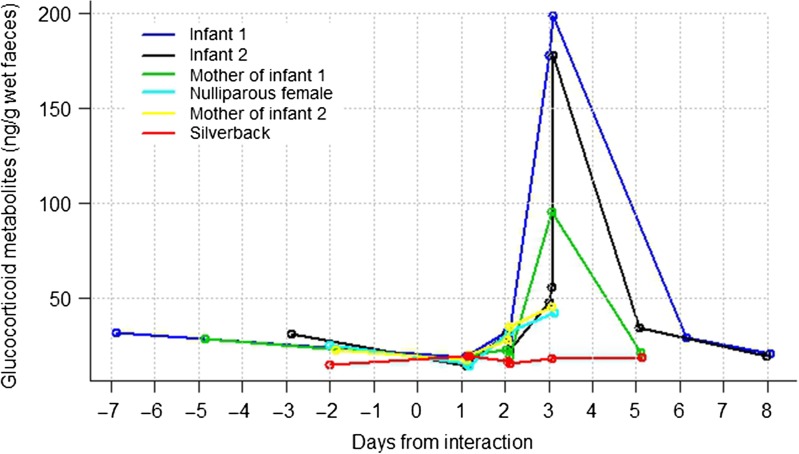
Faecal glucocorticoid metabolite concentration profiles of individual gorillas before and after an inter-unit interaction in six animals from Inshuti group.

In Inshuti group, five of the six gorillas had elevated FGM concentrations post-event (absolute mean 57 ± 54.5 ng/g wet faeces) relative to their pre-event FGM level (that is, the last FGM sample collected before the interaction event; Fig. 2). Across animals, FGM peaked ~3 days after the interaction and returned to pre-event concentrations by day 5 or 6. However, we were unable to evaluate faeces from post-event day 4. The two infants in the group showed the largest FGM elevation, with an 8- and 7.5-fold increase, respectively, followed by the mothers of the infants and the nulliparous female (Fig. 2). The silverback's FGM concentration remained at this pre-event level throughout. However, we were unable to obtain faecal samples from the silverback during post-event days 3–5, so we may have missed samples with elevated FGM concentrations.

#### Inter-unit interaction 2

**Figure 3:**
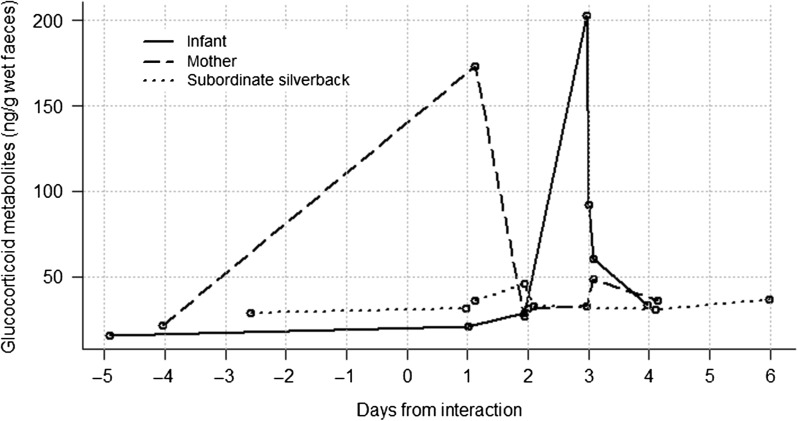
Faecal glucocorticoid metabolite concentration profiles of three individual gorillas from Ugenda group before and after an interaction with a solitary silverback. The infant lost a finger in the interaction.

Faecal samples were collected from the injured infant, her mother and the subordinate silverback; that is, the three animals that were primarily involved in the interaction. The injured infant and mother had clearly elevated FGM concentrations after the interaction (69.7 ± 61.9 ng/g wet faeces), up to 8.8- and 7.3-fold above pre-event levels, respectively (Fig. 3). The mother's FGM concentration peaked shortly after the interaction day (27 h post-event), and fell to pre-event concentration on day 2. The infant's FGM peaked on day 3 and then declined steeply, nearly back to her pre-event value within a few hours after the peak. The subordinate silverback's FGM peaked at 2-fold above the pre-event level (absolute elevation 46.2 ng/g wet faeces) around day 2, then returned nearly to the pre-event level (absolute value 32.6 ng/g wet faeces).

#### Inter-unit interaction 3

**Figure 4:**
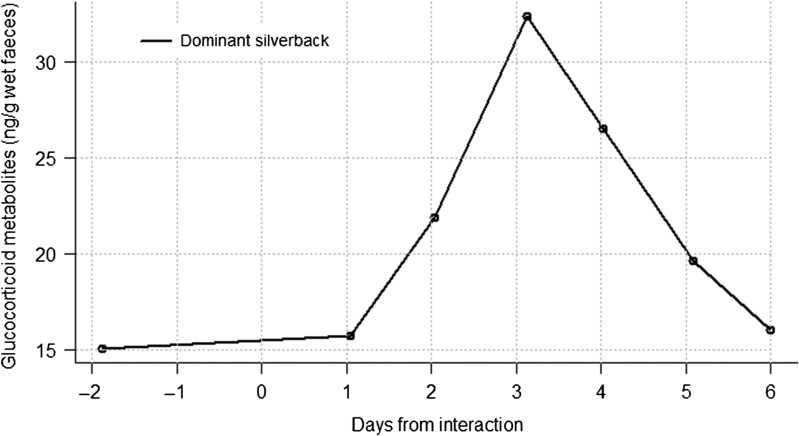
Faecal glucocorticoid metabolite concentration profile of the dominant silverback from Kuryama group before and after an interaction with a solitary silverback.

Kuryama group's dominant silverback had an elevated FGM concentration that peaked at 1.6-fold above the pre-event level between days 3 and 4, with a mean (absolute value) elevated FGM of 26.9 ± 5.3 ng/g wet faeces (Fig. 4). The elevation lasted until day 6, when the pre-event value was reached again.

#### Inter-unit interaction 4

**Figure 5:**
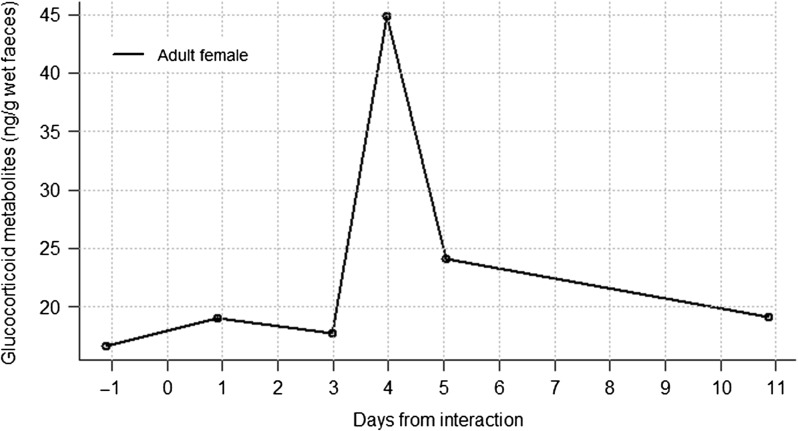
Faecal glucocorticoid metabolite concentration profile of an adult female in Kuryama group before and after an interaction with Inshuti group.

The adult female's post-event FGM level peaked with a 2.2-fold increase at day 3 after the interaction, followed by a steep decline at day 5 (Fig. 5). Her absolute mean elevated FGM concentration was 34.5 ± 14.7 ng/g wet faeces after the interaction.

### Time gap of elevated FGM concentrations post-events

**Figure 6:**
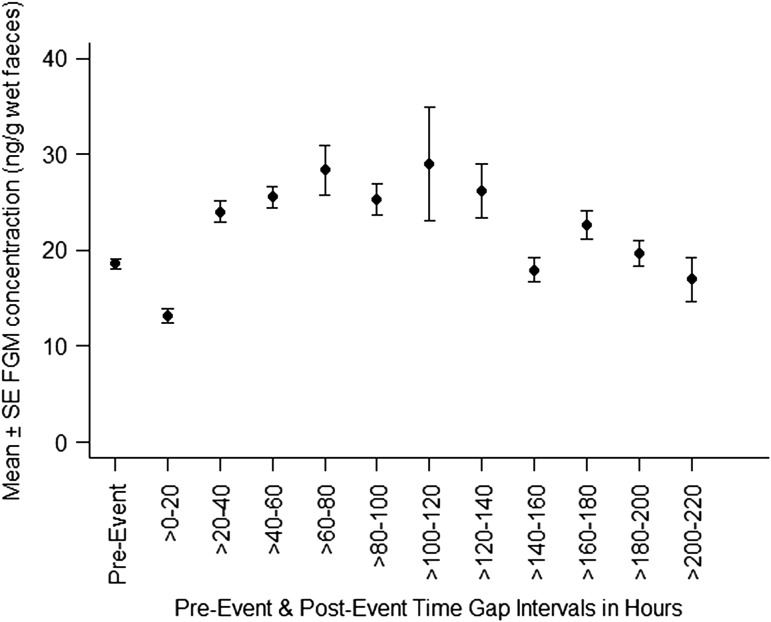
Mean (±SEM) of faecal glucocorticoid metabolite (FGM) concentration (removal of the two highest values above 150 ng/g wet faeces to adjust scale) of pre-event samples and post-event samples, collected >0–220 h after inter-unit interactions.

Individuals exhibiting elevated FGM concentrations after inter-unit interactions were from all age–sex classes (Table 1). The distribution of sample points suggested it was most appropriate to divide data into 20 h intervals rather than 24 h intervals for analysis. There was a significant elevation of FGM concentrations from pre-event samples between 20 and 140 h after events (Table 2 and Fig. 6). The peak most often occurred on day 3, between 60 and 80 h after an interaction.

**Table 2:** Mixed-effects model parameter estimates showing the relationship between faecal glucocorticoid metabolite concentrations and excretion lag times (presented in 20 h intervals) after inter-unit interactions

Time gap intervals (h)	b ± SE	*t*	*P*-value
>0–20	−0.002 ± 0.059	−0.531	0.596
>20–40	0.260 ± 0.069	3.788	**0.002**
>40–60	0.288 ± 0.068	4.202	**<0.001**
>60–80	0.371 ± 0.073	5.076	**<0.001**
>80–100	0.332 ± 0.089	3.725	**<0.001**
>100–120	0.381 ± 0.146	2.607	**0.010**
>120–140	0.353 ± 0.108	3.268	**0.001**
>140–160	−0.068 ± 0.121	−0.561	0.575
>160–180	0.226 ± 0.130	1.739	0.084
>180–200	0.032 ± 0.250	0.130	0.897
>200–220	−0.162 ± 0.178	−0.910	0.364

Reference level is the last sample from each animal in the week leading up to the event. *n* = 233 samples from 25 inter-unit interactions involving 34 gorillas; *F* = 5.69, d.f. = 11, *P* < 0.001. Significant effects at *P* = .05 are bold.

## Discussion

### Biological validation

This study used a field-friendly faecal hormone metabolite extraction method (Santymire and Armstrong, 2010; Murray *et al*., 2013) successfully to measure and validate FGM as a measure of stress in endangered Virunga mountain gorillas. We detected cause–effect relationships between inter-unit interactions and increased FGMs with elevations up to 8.8-fold above pre-interaction concentrations, indicating that such social events can be extremely stressful. In primates, inter-unit interactions can result in female transfers, male takeovers and severe injuries or death, but so far elevated FGM concentrations following such events have been reported only in adult male white-faced capuchins (*Cebus capucinus*; Schoof and Jack, 2013) and adult male and female chacma baboons (*Papio hamadryas ursinus*; Beehner *et al*., 2005; Bergman *et al*., 2005; Engh *et al*., 2006). To our knowledge, this is the first study demonstrating that this natural event can also cause a physiological stress response in juveniles and infants, who are vulnerable to the threat of infanticide (Fossey, 1984; Watts, 1989; Sicotte, 1993; Robbins *et al*., 2013).

There was strong inter- and intra-individual variation in elevated FGM levels after inter-unit interactions, demonstrating that individual mountain gorillas are affected differently by the same event. A comprehensive investigation is needed to determine what factors explain such variation (e.g. length of interactions, group composition, relatedness between interacting groups, presence of cycling females, severity of wounding, vulnerability of individuals during interactions). Since 2007, the annual interaction rate in the study population has increased 6-fold as a result of continuing population growth in an isolated forest island, resulting in more groups and a higher group density (Caillaud *et al*., 2014). The long-term impact of increasing exposure to these stressful events may have lasting effects on the reproduction, health and, thus, survival of the Virunga mountain gorillas, and needs to be monitored closely. The biological validation described here paves the way for future studies examining the relationship between adrenocortical activity and a variety of social, environmental and human-induced potential stressors for this gorilla population.

### Time gap between event and stress response

Our results revealed significant elevation of FGM from pre-event levels until 20–140 h after inter-unit interactions, with the peak most often occurring 3 days post-event. This elevation is comparable to FGM analysis from captive western lowland gorillas following adrenocorticotrophic hormone challenge tests (Heistermann *et al*., 2006; Nizeyi *et al*., 2011a; Shutt *et al*., 2012) and transportation between zoos for breeding purposes (e.g. an adult female showed a 7.6-fold increase in FGM; Jacobs *et al*., 2014). Individual FGM profiles before and after inter-unit interactions showed that elevated FGM concentrations can quickly drop towards pre-event levels, implying that intensive faecal collection, especially around day 3 post-event, is crucial to assess the magnitude of any stressor.

### Individual differences and biotic and environmental effects

Basal FGM concentrations varied between adult male and female gorillas. Males excreted lower levels than females, replicating results from a study in captive animals involving one adult male and one female western lowland gorilla (Jacobs *et al*., 2014). There are three potential explanations for this difference. Adult females may indeed be more vulnerable to stress, but alternatively, the differences might reflect higher energetic demands and physiological differences related to female reproductive states. For female mammals, reproductive state can be another source of inter- and intra-individual differences in basal FGM concentrations (chacma baboons, *P. hamadryas ursinus*, Weingrill *et al*., 2004; baboons, Gesquiere *et al*., 2008; chimpanzees, Thompson *et al*., 2010; rhesus macaques, *Macaca mulatta*, Hoffman *et al*., 2010). For example, higher FGM concentrations were associated with pregnancy in chacma baboons (Weingrill *et al*., 2004) and ring-tailed lemurs (*Lemur catta*; Cavigelli, 1999) and with lactation in spotted hyenas (Goymann *et al*., 2001).

Finally, there may also simply be species-specific sex differences in hormone metabolism and excretion (see Touma *et al*., 2003; Touma and Palme, 2005; Goymann, 2012). Other authors have proposed that biological and physiological validation of hormone metabolite measures should be conducted for both sexes separately (Touma and Palme, 2005; Goymann, 2012). Based on the present results, we can conclude that the field and laboratory methods applied in this study allow detection of cause–effect relationships in both sexes, but direct comparisons between male and female FGM concentrations in mountain gorillas may not be meaningful except when comparing relative changes in FGM concentrations.

Individual variation in basal FGM concentrations also occurred within adult males and females, which might be driven by social rank. An association between social rank and GC concentrations has been demonstrated in many primate species (olive baboons, *Papio anubis*, Sapolsky, 1982; Japanese macaques, *Macaca fuscata*, Barrett *et al*., 2002; review by Goymann and Wingfield, 2004; baboons, *Papio cynocephalus*, Gesquiere *et al*., 2011; review by Creel *et al*., 2013; chimpanzees, *Pan troglodytes schweinfurthii*, Markham *et al*., 2013) and other social mammals, birds and fish (reviews by Goymann and Wingfield, 2004; Creel *et al*., 2013). Future studies need to explore factors that are associated with individual variation in both sexes of wild mountain gorillas.

Consistent with results from the Bwindi mountain gorilla population (Nizeyi *et al*., 2011b) and other species with long gut passage times and slow FGM excretion, our data showed no circadian patterns. This may be because of complete dilution of pooled faeces in the digestive tract before excretion (Millspaugh and Washburn, 2004). However, our results did indicate variation in FGM concentrations between collection months, which may point to temporal changes in basal FGM concentrations and needs to be investigated further. In primates, seasonal cortisol variation is associated with multiple factors, which are often interrelated and difficult to disentangle. Temporal GC production in the Virunga gorilla population might be associated with variation in rainfall and temperature, which determines the four main seasons (long and short wet season and long and short dry season; for similar examples in other primates, see Beehner and McCann, 2008; Gesquiere *et al*., 2008; Foerster *et al*., 2012). The Virunga mountain gorillas’ habitat covers a wide altitudinal range, from approximately 2200 to 3800 m (McNeilage, 2001). Different altitudes have different rainfall and temperatures and contain different types of vegetation, which leads to changes in diet composition and thus may also contribute to temporal GC changes (Beehner and McCann, 2008; Goymann, 2012). Annual fluctuations in the number of tourist visits and the occurrence of illegal activities (e.g. snares in the home ranges of the study groups) might also be associated with temporal variation in stress levels. To increase our knowledge of the Virunga mountain gorilla stress physiology and inter-individual differences more generally, future research also needs to examine age-related patterns, plus social factors such as dominance rank, group size and group composition.

This study provides the foundation—a biologically validated, field-friendly faecal hormone metabolite extraction and laboratory EIA analysis method (Santymire and Armstrong, 2010; Murray *et al*., 2013)—for non-invasive monitoring of stress in the endangered Virunga mountain gorilla population. In future studies, this method can be used to examine the relationship between animal population growth, increasing anthropogenic pressure, and stress physiology in this critically endangered ape. The integration of a long-term stress-monitoring programme into existing research practices will provide crucial information for decision-makers in the conservation community, which will assist in answering important conservation questions and developing effective interventions.
